# Sanguinaria

**Published:** 1889-02

**Authors:** 


Sanguinaria: Neuralgia in upper jaw extending to nose, eye, ear, neck, and side of head; shooting, burning pains; must kneel down and hold head tightly to the floor.

